# Effectiveness and safety of PD-1/PD-L1 inhibitors in the treatment of solid tumors: a systematic review and meta-analysis

**DOI:** 10.18632/oncotarget.18316

**Published:** 2017-05-31

**Authors:** Xiaohui Wang, Zhengqiang Bao, Xiaoju Zhang, Fei Li, Tianwen Lai, Chao Cao, Zhihua Chen, Wen Li, Huahao Shen, Songmin Ying

**Affiliations:** ^1^ Department of Respiratory and Critical Care Medicine, Second Affiliated Hospital, Institute of Respiratory Diseases, Zhejiang University School of Medicine, Hangzhou, China; ^2^ Department of Pharmacology, Zhejiang University School of Medicine, Hangzhou, China; ^3^ Department of Respiratory Medicine, Zhengzhou University People's Hospital, Henan Provincial People's Hospital, Zhengzhou, China; ^4^ State Key Laboratory of Respiratory Diseases, Guangzhou, China

**Keywords:** PD-1, PD-L1, nivolumab, pembrolizumab, cancer

## Abstract

**Background:**

PD-1/PD-L1 inhibitors have been implicated as potentially effective anti-cancer therapies. Some clinical randomized controlled trials (RCTs) have been completed for a variety of PD-1/PD-L1 inhibitors to treat various malignancies, and more RCTs are still under way. We carried out this systematic meta-analysis to evaluate the efficacy and safety of PD-1/PD-L1 inhibitors in the treatment of solid tumors.

**Methods:**

We searched PubMed, EMBASE, clinical trial registers, conference reports, and related reviews. Eligible RCTs that compared PD-1/PD-L1 inhibitors with other chemotherapy agents or placebo in solid tumor patients were included. For each RCT, progression-free survival (PFS), overall survival (OS), objective response rate (ORR), disease control rate (DCR), stable disease rate (SDR), progressive disease rate (PDR), and adverse events (AEs) were pooled for meta-analysis.

**Findings:**

Based on an analysis of 10 eligible RCTs, PD-1/PD-L1 inhibitors were found to significantly improve PFS (Hazard ratio (HR), 0.65; 95% confidence interval (CI) 0.53 to 0.79, *P*<0.001), OS (HR, 0.69; 95%CI 0.62 to 0.76, *P*<0.001), and ORR (Risk Ratio (RR) 292; 95% confidence interval (CI) 2.06 to 4.15, *P*<0.00001) in all populations, including melanoma and NSCLC subgroups. However, they failed to increase the DCR of cancer patients (RR 1.15; 95%CI 0.91 to 1.45, *P*=0.25). Furthermore, less AEs were observed in the PD-1/PD-L1 inhibitor groups than the control groups.

**Interpretation:**

PD-1 inhibitors are more effective for improving the PFS, OS, and ORR of cancer patients with little toxicity, despite having little effect on increasing of the DCR.

## INTRODUCTION

Escape from immune surveillance is an important characteristic of carcinoma [1]. The development of therapies to enhance tumor immunity has become an important target for cancer treatment strategies [2]. Immune checkpoint inhibitors have achieved remarkable progress in tumor treatment, and two vital checkpoint receptors, CTLA-4 and the programmed death-1 receptor (PD-1), have been studied most extensively in clinical cancer immunotherapy [3, 4]. These receptors play significant roles in regulating the immune response against malignancy.

The CTLA-4 blocking antibody ipilimumab [3] has entered clinical trials for the treatment of different human cancers. PD-1 blocking antibodies have also been studied extensively. PD-1 is a cell surface receptor that belongs to the CD28 immunoglobulin superfamily, which is usually expressed on activated T cells, Tregs, activated B cells, NK cells, and monocytes [4], [5]. PD-1 is an immune checkpoint that plays a significant role in down-regulating the immune system by limiting the activity of T-cells in the periphery during an inflammatory response [6]. The checkpoint receptor PD-1 interacts with its ligands PD-L1 and PD-L2 to inhibit T cell activation and proliferation, thereby promoting immunological self-tolerance [7, 8].

Tumor cells often use the PD-1-PD-L1/2 pathway to evade immune-cell attack [9]. Blockade of this pathway was shown to restore and improve antitumor immune response. In cancer patients, PD-1 is usually highly expressed on T cells and causes tumor-related immune suppression [10]. It has recently emerged as an effective therapeutic option for various cancers, with anti-PD-1 or anti-PD-L1 antibodies showing broad-ranging anti-tumor activity in early-phase trials [11–13].

Notably, the anti-PD-1 antibodies pembrolizumab and nivolumab were approved by the US FDA for the treatment of patients who were previously treated for metastatic melanoma [14]. Nivolumab is a monoclonal antibody against PD-1 [15] and has been tested in trials for the treatment of melanoma, non-small cell lung cancer (NSCLC) [16], ovarian cancer, and renal cell carcinoma [17]. Pembrolizumab is a monoclonal antibody that blocks the interaction of PD-1 on T cells with its ligands [18], which is suggested for antitumor activity in patients with advanced NSCLC or advanced melanoma. In addition, a series of phase I/II trials using pembrolizumab on other types of cancer is currently being investigated. Other anti-PD-1 antibodies and anti-PD-L1 antibodies are also being tested in different clinical trials, such as pidizumab [19], MPDL3280A [20], and BMS-936559 [12].

Recent studies have shown that anti-PD-1 and anti-PD-L1 monoclonal antibodies play positive roles in the development of cancer treatment. So far, a number of phase 2 or 3 studies have been completed on PD-1 blockade for different tumor treatments, and clinical trials for PD-L1 inhibitors are still in progress. Thus, we performed a meta-analysis that incorporates all available results to evaluate the efficacy and safety of PD-1 inhibition therapy.

## RESULTS

**Figure 1:**
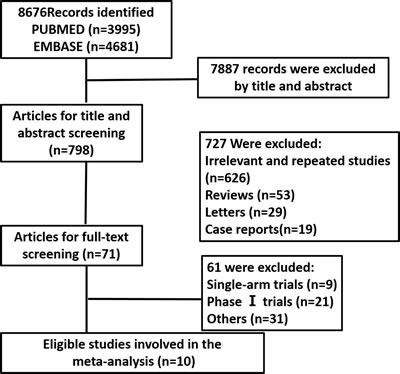
The process of literature search and eligible trials selection N: the number of studies.

Our search strategy originally retrieved 8676records. Among these, 7887articles were excluded for not being RCTs, and 798 articles were excluded by screening the title and abstract. After carefully reading the full texts of the remaining 71 articles, 10 eligible studies [21–30] met the inclusion criteria, as shown in Figure 1. Finally, a total of 5246 patients were enrolled. The median age of the patients was similar and ranged from 59 to 66 years. The 10 included studies were all published between 2014 and 2016. Of the 10 included studies, 7 studies used the PD-1 inhibitor nivolumab, 2 studies used the PD-1 inhibitor pembrolizumab, and 1 study investigated the PD-L1 inhibitor atezolizumab. There were 6 studies [23, 26–30] [23, 26–30] [25, 28–32] [25, 28–32] [25, 28–32] about melanoma, 3 studies were related to NSCLC treatment, and 1 study was on renal-cell carcinoma. The detailed characteristics of the 10 studies are presented in Table 1.

**Table 1:** Characteristics of the trials included in the meta-analysis

Characteristics of Patients in Constituent Trials								
Research orientation	Phase	Cancer type	Treatment	Median PFS (months)	Female/Patients(No.)	Age in years, Madian(range)	PD-L1 positive/ negative	BRAF mutation/ wild-type
Weber J.S 2015	III	melanoma	Nivolumab 3mg/kg every 2 weeks	4.7	96(272)	59(23-88)	134/138	60/212
			Chemotherapy	4.2	48(133)	62(29-85)	29/66	29/104
Larkin.J 2015	III	melanoma	Nivolumab 3mg/kg every 2 weeks	6.9	114(316)	59(25-90)	80/208	100/316
			Nivolumab 1mg/kg every 3 weeks + Ipilimumab 3mg/kg every 2 weeks	11.5	108(314)	59(18-88)	68/210	101/213
			Ipilimumab	2.9	113(315)	61(18-89)	75/202	97/218
Robert.C 2015	III	melanoma	Pembrolizumab 3mg/kg every 2 weeks	5.5	118(279)	61(18-89)	225/54	98/181
			Pembrolizumab 3mg/kg every 3 weeks	4.1	103(277)	63(22-89)	221/56	97/180
			Ipilimumab	2.8	116(278)	62(18-88)	225/53	107/171
Brahmer.J 2015	III	NSCLC	Nivolumab 3mg/kg every 2 weeks	3.5	24(135)	62(39-85)	NA	NA
			Docetaxel	2.8	49(137)	64(42-84)	NA	NA
Robert.C 2014	III	melanoma	Nivolumab 3mg/kg every 2 weeks	5.1	89(210)	64(18-86)	74/136	0/202
			Dacarbazine	2.2	83(208)	66(26-87)	74/134	0/204
Ribas.A 2015	II	melanoma	Pembrolizumab 2mg/kg every 2 weeks	5.4	76(180)	62(15-87)	NA	44/136
			Pembrolizumab 10mg/kg every 3 weeks	5.8	72(181)	60(27-89)	NA	40/141
			Chemotherapy	3.6	65(179)	63(27-87)	NA	41/138
Postow MA 2015	I	melanoma	Nivolumab 1mg/kg every 3 weeks + Ipilimumab 3mg/kg every 2 weeks	NA	32(95)	64(27-87)	NA	23/72
			Ipilimumab	4.4	15(47)	67(31-80)	NA	10/37
Borghaei.H 2015	III	NSCLC	Nivolumab 3mg/kg every 2 weeks	2.3	141(292)	61(37-84)	NA	NA
			Docetaxel	4.2	122(290)	64(21-85)	NA	NA
Motzer R.J 2015	III	Renal-cell Carcinoma	Nivolumab 3mg/kg every 2 weeks	4.6	95(410)	107(411)	NA	NA
			Everolimus	4.4	62(411)	62(18-86)	NA	NA
Fehrenbacher.L 2016	II	NSCLC	Atezolizumab 1200mg/m^2^ every 3weeks	2.7	51(144)	62(42-82)	96/48	NA
			Docetaxel	3	67(143)	61(36-84)	82/61	NA

NSCLC: Non-Small-Cell Lung Cancer; NA: not available; PFS: Progression-free survival

### Progression-free survival (PFS), overall survival (OS), objective response rate (ORR), disease control rate (DCR), stable disease rate (SDR), and progressive disease rate (PDR) of all populations

PFS, OS, ORR, DCR, SDR and PDR are the important end points of tumor RCTs. Progressive-free survival (PFS) is a measure of the activity of a treatment on tumors. It is the time that passes from a certain date (generally the first day of treatment, or the day in which a patient is enrolled in a clinical trial) and the date on which disease “progresses” or the date on which the patient dies, from any cause. Overall survival (OS) is a primary end point usually, Patients with tumors can die directly from that disease or from an unrelated cause. When the precise cause of death is not specified, this is called the overall survival rate or observed survival rate. Researchers often use mean overall survival rates to estimate the patient's prognosis. Objective response rate (ORR) is another important end point of clinical cancer research, Objective Response Rate is the percentage of patients whose cancer shrinks or disappears after treatment. Which often used as a clinical endpoint for clinical trials of cancer treatments. Disease Control Rate (DCR) is the sum of complete response rate, partial response rate and stable disease rate. Progressive Disease Rate (PDR) is the percentage of patients whose cancer progress.

**Figure 2:**
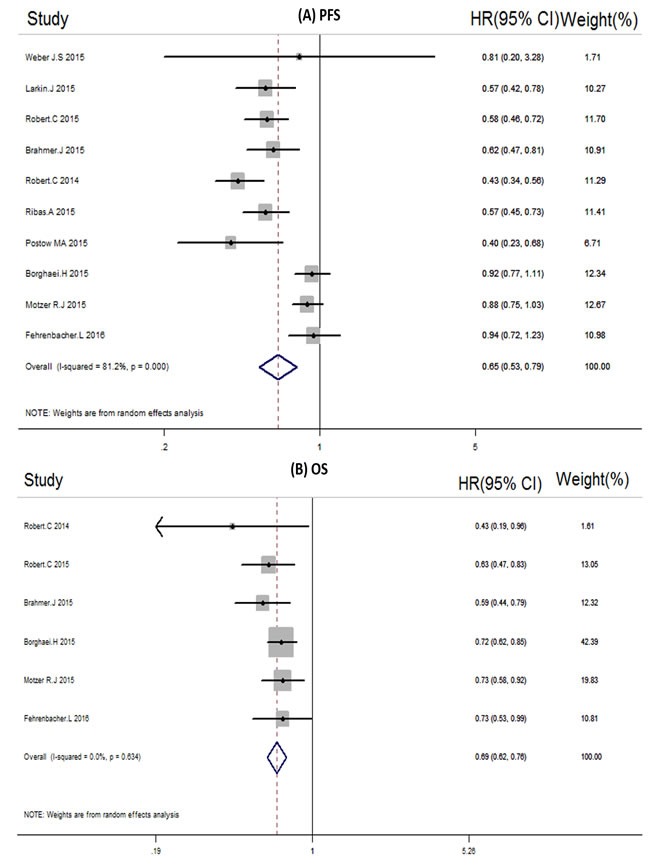
**A.** Forest plots of the pooled Hazard ratios (HRs) of Progressive-free survival (PFS) PFS in overall population. **B**. Forest plots of the pooled Hazard ratios (HRs) of Overall survival in overall population.

There were 10 trials that reported the PFS, and 6 trials reported the OS of the overall population. The PFS of patients treated with PD-1/PD-L1 inhibitor was significantly greater than those of the control arms with an HR of 0.65 (95% CI 0.53 to 0.79) (Figure 2A). The statistical analysis of OS based on 6 RCTs revealed that the PD-1/PD-L1 inhibitor significantly improved the OS of cancer patients compared with the control (HR, 0.69; 95% CI 0.62 to 0.76) (Figure 2B). As mentioned, 6 RCTs were related to melanoma and 3 RCTs were related to lung cancer, we set the subgroup analysis of PFS in different cancer types and different drug types (Table 2). From the results, PD-1 inhibitors do better in melanoma with an HR of 0.53(0.46 to 0.60) than NSCLC with an HR of 0.82(0.64 to 1.05), The results of OS subgroup analysis, PD-1 inhibitors not only improved the OS in melanoma patients with HR 0.60( 0.46 to 0.79) but also improved the OS in NSCLC patients with HR 0.70( 0.61 to 0.79) .

**Table 2:** Summary results of the pooled HRs for PFS and OS by subgroup analysis

		Pooled PFS				Pooled OS			
		No.of trials	HR(95%CI)	I^2^	*P*	No.of trials	HR(95%CI)	I^2^	*P*
Cancer type	melanoma	6	0.53(0.46,0.60)	5.00%	0.385	2	0.60(0.46,0.79)	0.00%	0.383
	NSCLC	3	0.82(0.64,1.05)	68.80%	0.041	3	0.70(0.61.0.79)	0.00%	0.474
	Renal-cell Carcinoma	1	0.88(0.75,1.03)	0.00%	NA	1	0.73(0.58,0.92)	0.00%	NA
Drug type	Nivolumab	7	0.63(0.48,0.83)	83.90%	0.000	4	0.69(0.62,0.78)	0.00%	0.405
	pembrolizumab	2	0.58(0.49,0.68)	0.00%	0.918	1	0.63(0.47,0.83)	0.00%	NA
	Atezolizumab	1	0.94(0.72,1.23)	0.00%	NA	1	0.73(0.53.0.99)	0.00%	NA

NSCLC: Non-Small-Cell Lung Cancer; PFS: Progression-free survival; OS: Overall survival; HR: Hazard ratio; CI: Confidence interval; *P*: P-value of Q-test for heterogeneity test. NA: not available.

**Table 3:** Relative risks with 95% confidence intervals for common adverse events (Grade≥3)

	Grade≥3					
Adverse event	No. of trials	Subjects	RR[95% CI]	*P*	*I*^2^ (%)	*P*^b^
Alopecia	2	309/300	0.32(0.03,3.10)	0.33	0	0.99
Anaemia	5	1270/1067	0.18(0.10,0.34)	<0.00001	0	0.95
Arthralgia	3	724/529	0.31(0.06,1.75)	0.19	0	0.59
Asthenia	5	1080/1029	0.28(0.09,0.88)	0.03	0	0.75
Colitis	3	685/613	0.35(0.05,2.67)	0.31	88	0.0002
Constipation	2	362/148	0.44(0.04,3.11)	0.5	12	0.29
Decreased appetite	4	1137/1105	0.27(0.06,1.18)	0.08	0	0.68
Diarrhoea	8	1755/1488	0.58(0.35,0.93)	0.03	0	0.56
Fatigue	9	2161/1885	0.40(0.19,0.83)	0.01	41	0.1
Nausea	8	1955/1680	0.31(0.12,0.80)	0.02	0	0.92
Neutropenia	3	596/568	0.02(0.00,0.09)	<0.00001	0	0.42
Prutirus	3	1496/989	0.44(0.10,1.83)	0.26	6	0.37
Rash	6	1428/1344	0.58(0.23,1.48)	0.26	0	0.42
dyspnea	3	813/754	2.02(0.51,8.00)	0.32	0.31	0.85
leukopenia	2	309/300	0.14(0.03,0.77)	0.02	0.3	0.58
maculopapular rash	2	272/217	3.18(0.37,27.22)	0.29	0.01	0.93
hypophysitis	2	372/302	0.35(0.08,1.49)	0.16	0.26	0.61
headache	2	407/357	0.96(0.11,8.61)	0.97	0.8	0.37
peripheral neuropathy	2	309/300	0.16(0.02,1.36)	0.09	0.02	0.88
pneumonitis	2	372/302	0.65(0.10,4.36)	0.66	0.33	0.57
pyrexia	3	538/486	0.79(0.13,4.73)	0.79	1.59	0.46
thrombocytopenia	2	384/376	0.07(0.01,0.54)	0.01	0.16	0.69

RR: Relative risk; CI: Confidence interval; *P*b: *P*-value of Q-test for heterogeneity test.

**Figure 3:**
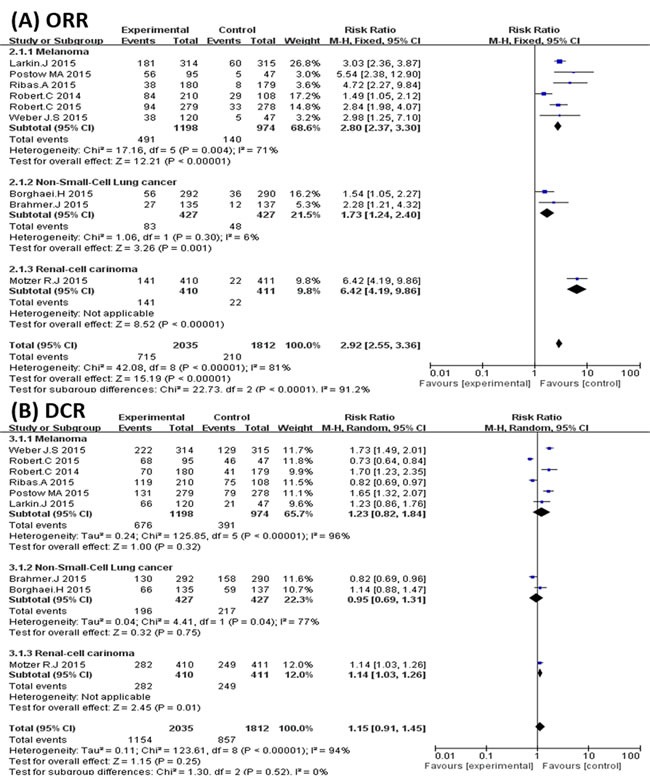
Subgroup analysis of cancer types **A**. Forest plots of the pooled Relative Risk (RR) of objective response rate (ORR); **B**. Forest plots of the pooled Relative Risk (RR) of disease control rate (DCR).

There were 9 trials that reported the ORR and DCR of the overall population among the 10 studies included. The ORR was also significantly higher in the PD-1 inhibitor treatment groups (715/2035, 35%) than the control groups (210/1812, 11%), with an RR of 2.92 (95% CI 2.06 to 4.15, *P* < 0.00001) (Figure 3A). Although the PD-1 inhibitor showed a slight trend of improving the DCR when compared with control arms, the result was not significant with an RR of 1.15 (95% CI 0.91 to1.45, *P* = 0.25) (Figure 3B).

**Figure 4:**
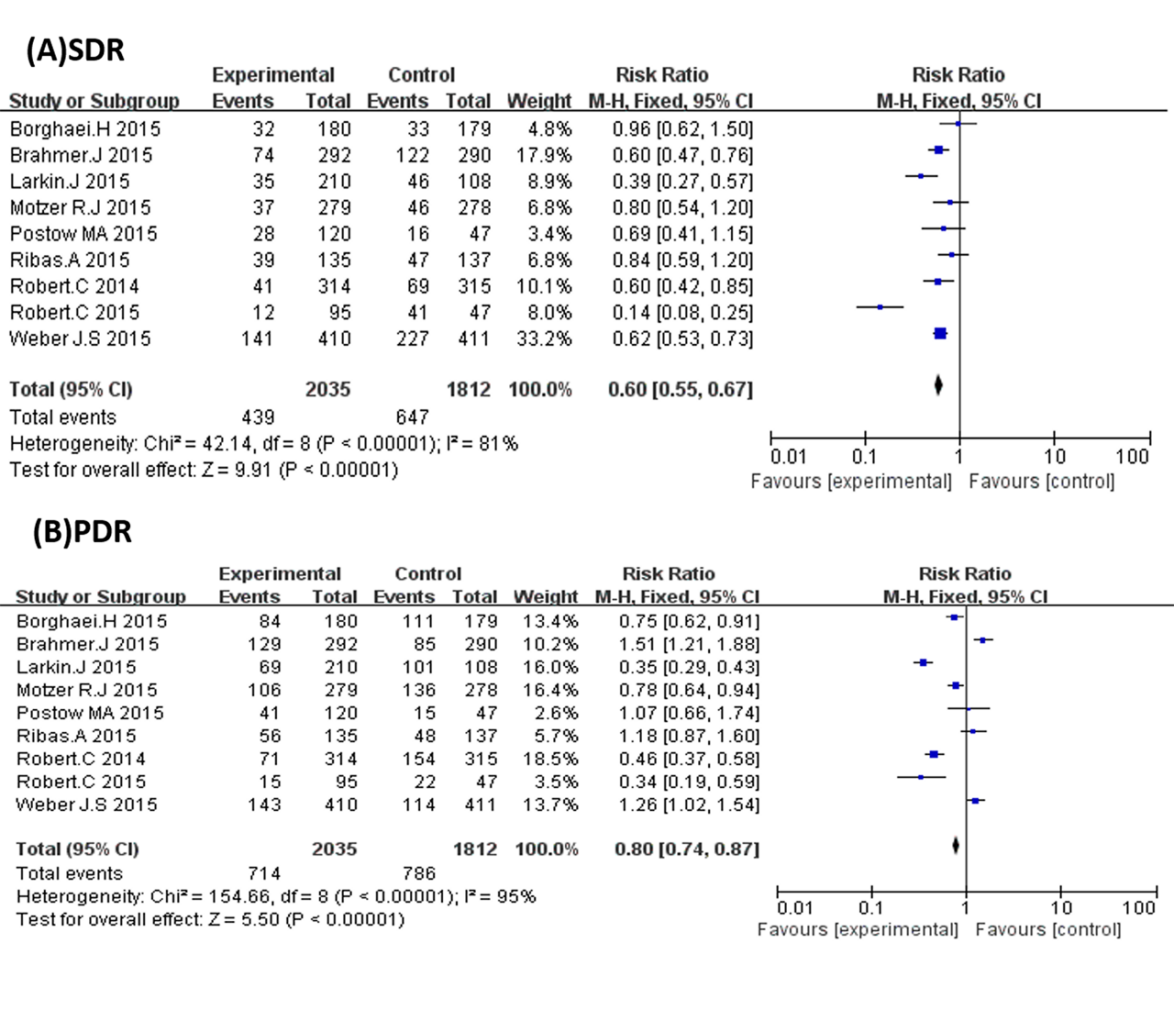
**A.** Forest plots of the pooled Relative Risk (RR) of stable disease rate (SDR) and **B.** Forest plots of the pooled Relative Risk (RR) progressive disease rate (PDR).

It was apparent that PD-1 inhibitors were more effective in improving the ORR of cancer patients. However, significantly more patients in the control arms reached stable disease status (RR 0.58; 95% CI 0.45 to 0.75; *P* < 0.0001) (Figure 4A). Although the difference was not significant, PD-1 inhibitors had a slight trend of decreasing the PDR compared with the control arms (RR 0.76; 95% CI 0.53 to 1.09; *P* = 0.13) (Figure 4B).

### Objective response rate (ORR) and disease control rate (DCR) of melanoma and NSCLC subgroups

As mentioned, 6 studies were related to melanoma and 2 studies were related to lung cancer. Thus, we carried out subgroup analyses to explore the efficiency of PD-1 inhibitors in the treatment of melanoma and lung cancer. The results show that PD-1 inhibitors could increase the ORR of melanoma patients compared with the control groups (RR 2.89; 95%CI 2.02 to 4.13; *P* < 0.00001) (Figure 3A). PD-1 inhibitors could also significantly increase the ORR of patients in the NSCLC populations (RR 1.72; 95%CI 1.22 to 2.43; *P* = 0.002) (Figure 3A). However, PD-1 inhibitors failed to increase the DCR of patients in both melanoma and NSCLC cancer populations (Figure 3B).

### Objective response rate (ORR) and disease control rate (DCR) of nivolumab and pembrolizumab subgroups

**Figure 5:**
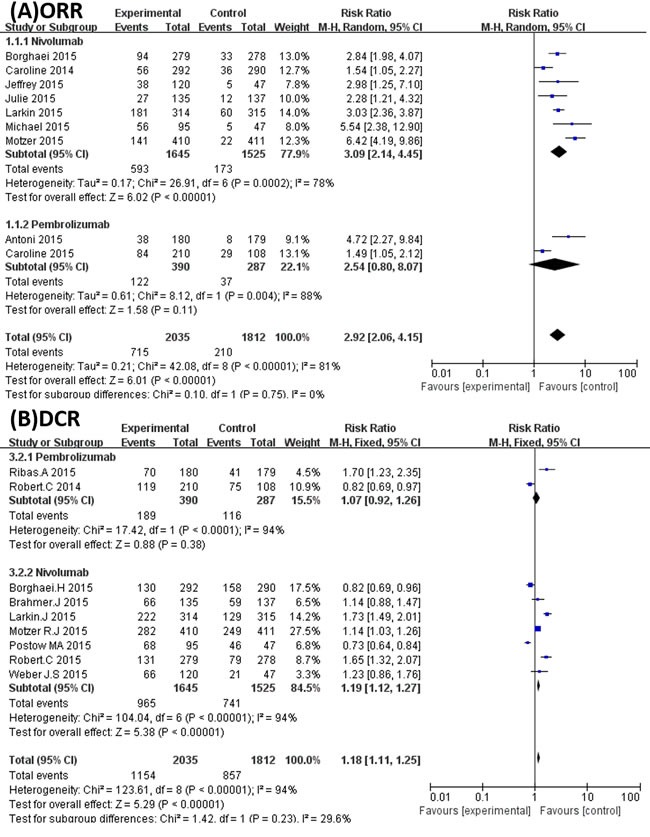
**A.** Forest plots of the pooled Relative Risk (RR) of objective response rate (ORR) in Subgroup analysis of nivolumab and pembrolizumab.; **B**. Forest plots of the pooled Relative Risk (RR) of disease control rate (DCR) in Subgroup analysis of nivolumab and pembrolizumab.

Our studies involved two kinds of PD-1 inhibitors: nivolumab (7 articles) and pembrolizumab (2 articles). The ORR was significantly higher in the nivolumab groups than in the control groups (RR 3.09; 95% CI 2.14 to 4.45; *P* < 0.00001) (Figure 5A). Although there was a similar trend in the pembrolizumab arms, the difference was not significant when compared with the control arms (RR 2.54; 95% CI 0.80 to 8.07; *P* = 0.11) (Figure 5A). However, in regard to DCR, both nivolumab and pembrolizumab produced no significant difference from the control groups (Figure 5B).

### Adverse events

**Figure 6:**
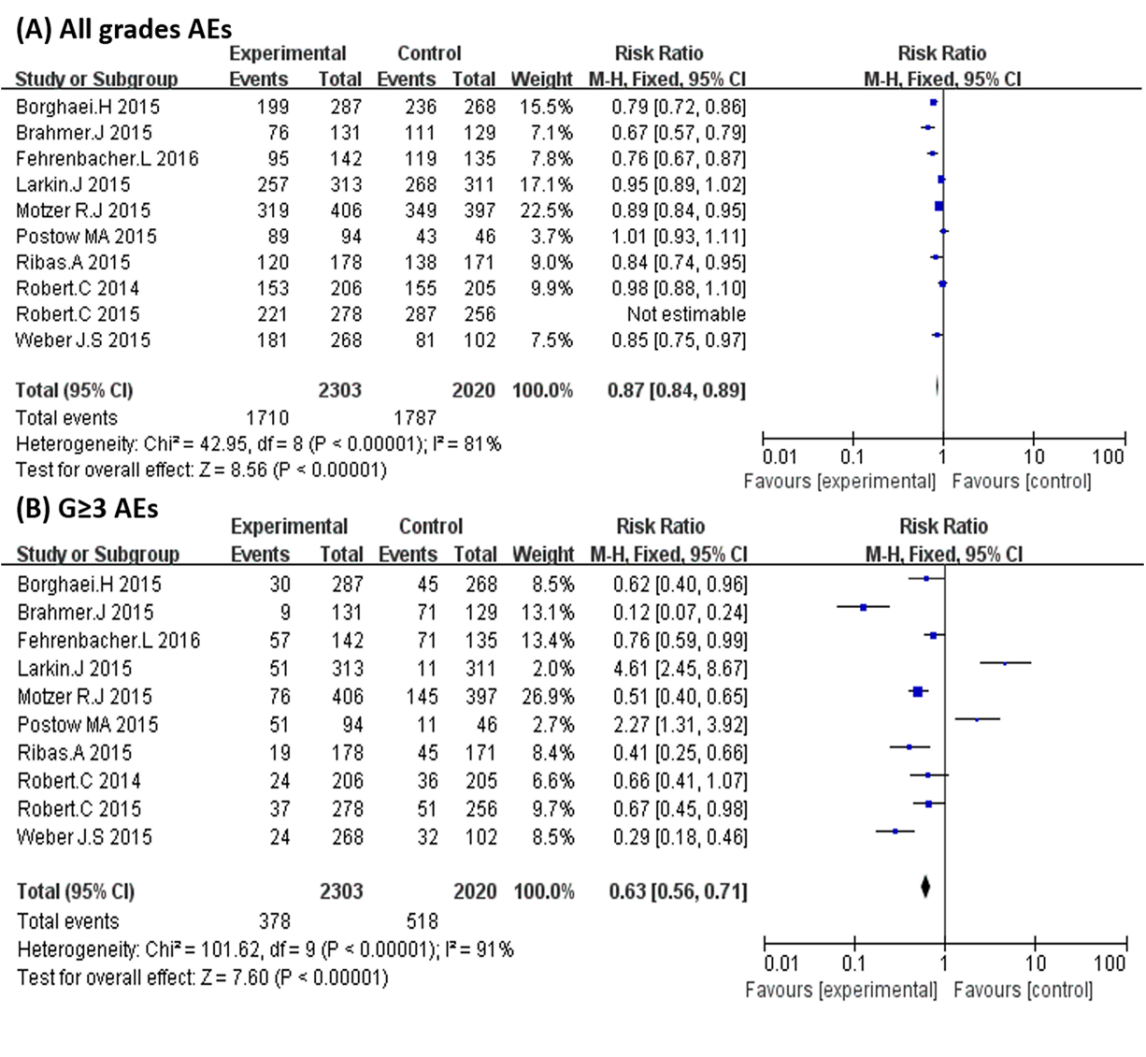
**A.** Relative Risks (RR) of common adverse events of all grades. **B**. Relative Risks (RR) of adverse events of grade ≥ 3.

In general, PD-1/PD-L1 inhibitors decreased AEs (1710/2303 for the PD1/PD-L1 inhibitor arms (74.3%) vs. 1787/2020 for the control arms (88.5%); *P* < 0.00001) (Figure 6A). This difference was more prominent in AEs with grade ≥ 3 (378/2161 of the PD-1/PD-L1 inhibitor arms (15.6%) vs. 518/2020 for the control arms (25.6%), *P* < 0.00001) (Figure 6B). The most common AEs (grade ≥ 3) that emerged in the RCTs were fatigue (reported in 10 studies), nausea (9 studies), diarrhea (9 studies), and rash (6 studies). When compared with the control arms, PD-1 inhibitors had low toxicity and could also decrease the risk of anemia, asthenia, diarrhea, fatigue, nausea, neutropenia, leukopenia, and thrombocytopenia (Table 2).

### Sensitivity analysis

**Figure 7:**
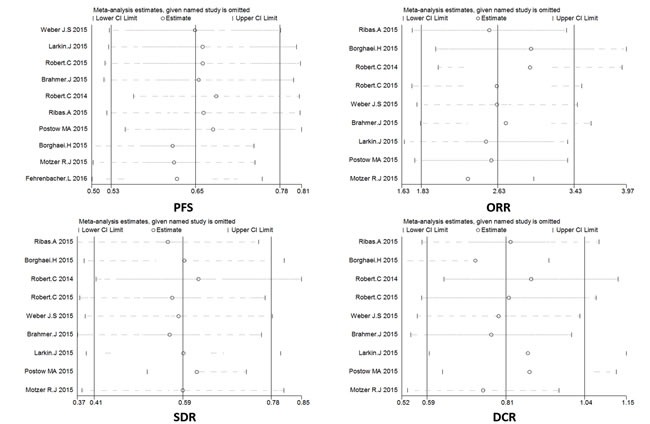
**A.** Sensitivity analysis of enrolled studies on progression-free survival (PFS). **B**. Sensitivity analysis of enrolled studies on objective response rate (ORR). **C**. Sensitivity analysis of enrolled studies on stable disease rate (SDR). **D**. Sensitivity analysis of enrolled studies on disease control rate (DCR).

Sensitivity analyses were carried out to evaluate the stability of the studies according to the effects of omitting each study. The sensitivity analysis results of PFS, ORR, SDR, and DCR showed that no individual study changed the pooled data overall, which suggests that our results are stable (Figure 7).

### Publication bias analysis

**Figure 8:**
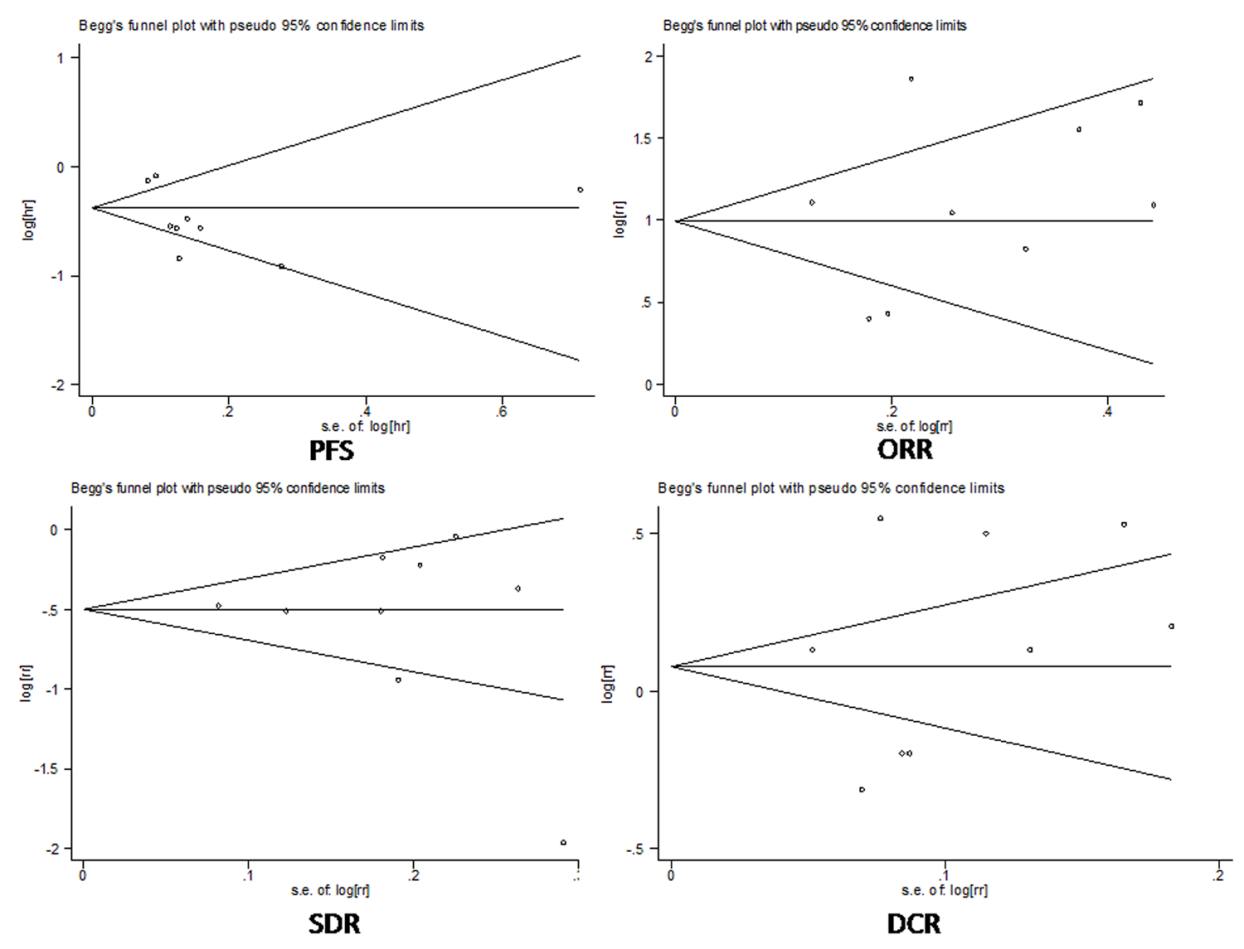
**A.** Funnel plot of publication bias on progressive-free survival (PFS). **B**. Funnel plot of publication bias on objective response rate (ORR). **C**. Funnel plot of publication bias on stable disease rate (SDR). **D**. Funnel plot of publication bias on disease control rate (DCR).

Begg's funnel plot and Egger's test were applied to assess the publication bias of the studies (Figure 8). The Z-value of Begg's test in the pooled analysis was 1.11 for PFS (*P* = 0.266), 0.73 for ORR (*P* = 0.466), 0.10 for SDR (*P* = 0.917), and 0.52 for DCR (*P* = 0.602). Egger's test showed that the bias of the meta-analyses was -1.85 for PFS (*P* = 0.114), 0.63 for ORR (*P* = 0.546), -0.72 for SDR (*P* = 0.494), and 0.63 for DCR (*P* = 0.549). The statistical results show that the bias from publications does not have a significant influence on the results of our meta-analysis.

## DISCUSSION

This meta-analysis included 10 RCTs and 5246 patients, and detailed data were extracted and calculated for PFS, OS, DCR, AEs, and other data. A comprehensive analysis was also done to evaluate the curative effect and safety of PD-1 inhibitors. PD-1 inhibitors presented a high curative effect on cancer patients. The PFS and OS of cancer patients treated with PD-1 inhibitors were improved significantly. In one of the trials [31], the median PFS of patients treated with PD-1 inhibitor was 11.5 months, while it was only 2.9 months for those treated without PD-1 inhibitor. It has been reported that a single dose of nivolumab could induce nearly a complete response in patients with cancer [32].

Our results also revealed that the ORR was 35% (715/2035) in PD-1 inhibitor treatment groups, while that in the control groups was only 11% (210/1812). The higher ORR also supported the longer PFS and OS in tumor patients treated with PD-1 inhibitors. Although the PD-1 inhibitors had a slight trend of increasing the disease control rate of cancer patients, it was not significant when compared with the control arms (1154/2035 for the PD-1 inhibitor arms (56.7%) *vs*. 857/1812 for the control arms (47.3%); *P* = 0.25).

The DCR included complete response (CR), partial response (PR), and stable disease (SD). The main reason for the discrepancy between PFS/OS/ORR and DCR may be that so many patients reached the stabile disease status in the control arms compared with the PD-1 arms that PD-1inhibitors produced weak increases in the DCR. On the other hand, PD-1 inhibitors had a slight trend of decreasing the PDR compared with control arms (RR 0.76; 95% CI 0.53 to 1.09; *P* = 0.13), although the difference was not significant. All of these results suggest that PD-1 inhibitors were better at improving the PFS, OS, and ORR of cancer patients, and they may have potential as powerful antitumor drug.

PD1/PD-L1 inhibitor drugs target immune checkpoints, and they may be very effective in the treatment of immune sensitive tumors. However, they may lack efficacy in some immune-insensitive cancers. The main cancers included in our research were melanoma (6 articles) and NSCLC (2 articles). Melanoma and NSCLC are immune-sensitive cancers and have the most abundant PD-L1 expression [33]. It has been reported that monoclonal antibodies against PD-1/PD-L1 interaction will prolong the OS in melanoma and NSCLC patients [34]. The FDA has approved the use of PD-1 inhibitors in the treatment of melanoma and NSCLC.

In this study, we evaluated the effect of PD-1 inhibitors on melanoma and NSCLC patients based on the ORR and DCR. Our results were consistent with previous reports, and PD-1 inhibitors showed a prominent effect in the treatment of melanoma and NSCLC. The ORR of patients treated with PD-1 increased significantly in the melanoma and NSCLC populations. PD-1 inhibitors are not limited to one tumor or tissue type [35]. In addition to melanoma and NSCLC, PD-1 inhibitors may also emerge as an effective antitumor strategy in various other common cancers, such as ovarian cancer, bladder cancer, and head and neck squamous cancer [30]. However, it seemed powerless to elevate the DCR of cancer patients in the present study. More effort is needed to clarify these results in the future.

It is well known that the immune system plays a vital role in antitumor activity. PD-1 is one of the important immune checkpoint receptors. In recent years, a series of drugs have been developed to target PD-1 checkpoint receptors, such as pembrolizumab, nivolumab, and pidilizumab [35]. Our study included two PD-1 inhibitors, nivolumab and pembrolizumab, which are monoclonal antibodies that bind to the PD-1 receptor and block its interaction with PD-L1 and PD-L2. This results in the release of the PD-1 pathway-mediated inhibition of the immune response, including the anti-tumor immune response [35, 36]. Both drugs were approved by the FDA for the treatment of cancers last year. We evaluated the antitumor effect of nivolumab and pembrolizumab, and the results showed that patients treated with these drugs had higher ORR (593/1645 (36%); 122/390 (31%)), which is in line with our expectations. However, the DCR of both nivolumab and pembrolizumab populations was not satisfactory. There was only one PD-L1 inhibitor in our study, and we had only enough data to compare anti-PD-1 and anti-PD-L1 immunotherapy.

The antitumor effect of PD-1 inhibitors is mainly due to the unrestrained T cell activation with immune checkpoint blockade, which may induce immune-related AEs. AEs like rash, fatigue, cough, diarrhea, constipation, and arthralgia were found in more than 20% of cancer patients receiving PD-1 inhibitors, but they were mainly grade 1 or 2 AEs and could be managed [36]. In this study, we pooled the total number of all grades of AEs, which showed that PD-1 inhibitors had an effect of decreasing AEs in all grade levels, especially AEs with grade ≥ 3. Traditional chemotherapeutics usually induce serious adverse events. However, our results revealed that PD-1 inhibitors have little toxicity and even have an effect of decreasing the risk of various AEs. In conclusion, PD-1 inhibitors are better for improving the PFS, OS, and ORR of cancer patients with little toxicity, but they are weak in increasing the DCR.

## MATERIALS AND METHODS

### Literature search and study selection

We carried out a comprehensive systematic retrieval for potential articles in the PubMed and EMBASE databases from inception to February 22th, 2017. The search terms used were “PD-1 inhibitors” or “PD-L1 inhibitors” or “Nivolumab” or “Pembrolizumab ” or “Atezolizumab” or “BMS936559” or “MPDL3280A” or “Durvalumab” or “Avelumab” or “MED14736”, combined with “Cancer” or “Tumor” or “Carcinoma”. The search was limited to clinical trials without restrictions on publication language. For comprehensive retrieval of articles, we searched relevant meeting abstracts, clinical trials in the clinical trial registry (http://www.clinialtrials.gov), and related review articles. To ensure the quality of eligible clinical trials, studies published with full text were included.

The following criteria were used for inclusion in the meta-analysis: randomized control trials (RCTs); the involvement of cancer patients; the use of PD-1/PD-L1 inhibitors alone or in combination with other agents for the treatment group along with placebo or other chemotherapeutic agents for the control group; at least one objective type of data reported, such as progression-free survival (PFS), overall survival (OS), objective response (including complete response and partial response), and adverse events. Studies were excluded in the following conditions: case reports; reviews; retrospective or prospective observational cohort studies; single-arm RCTs; and PD-1/PD-L1 inhibitors were used in both the experiment arms and control arms. When repeated studies were identified, the most elaborate and latest articles were included. Two investigators (Bao Z and Wang X) independently reviewed the articles for eligibility.

### Data extraction

This systematic review was conducted according to the PRISMA guidelines. For each enrolled RCT, the following details were extracted and presented: the first author's surname, journals, year of publication, number of participants, median age, sex (female *vs*. male), cancer type, clinical trial phase, treatment arm, median progression-free survival, BRAF status, and PD-L1 status. The following data were extracted for the systematic meta-analysis: progression-free survival (PFS) and overall survival (OS) (hazard ratio (HR) with 95% confidence interval (CI)), objective response (including complete response and partial response), stable disease and progressive disease, disease control rate (including complete response, partial response, and stable disease), and adverse events (AEs).

### Statistical analysis

All pooled data were analyzed with Stata version 12.0 (StataCorp, College Station, Texas) and Review Manager (version 5.2, The Cochrane Collaboration, Oxford, UK). All statistical tests were two-sided, and P≤0.05 was considered statistically significant. The HR and 95% CIs were used to assess the OS and PFS between the PD-1 inhibitor group and control group. Data on objective responses, stable disease, and adverse events were also pooled to calculate RR with 95% CIs. The degree of heterogeneity was measured by the *I*^2^ statistic [37] with *I*^2^ < 25%, 25-75%, and > 75% representing low, moderate, and high degrees of inconsistency, respectively.

We used a fixed-effect model if the heterogeneity was low in the analyses, and a random-effects model was applied otherwise. Subgroup analysis was also carried out according to the different tumor types and different PD-1 inhibitors. Sensitivity analysis was performed to observe the effect of a single study on the overall results. We used a Funnel plot and Egger's regression asymmetry test to identify the potential publication bias of the studies [38].
